# Supplementary material for: Photosynthetic Toxicity of Enrofloxacin on Scenedesmus obliquus in an Aquatic Environment
Source: Int J Environ Res Public Health. 2022 May 3;19(9):5545. doi: 10.3390/ijerph19095545 (PMC9105898; doi:10.3390/ijerph19095545)
Supplement: Supplementary file 1 [file ijerph-19-05545-s001.zip › ijerph-1679974-supplementary.pdf]

Toxicity effect of typical antibiotics on *Scenedesmus obliquus* in aquatic environment

Zhiheng Li[a, b], Xianghong Zhang[a, b], Hong Fang[a, b], Xinmi Dai [a, b], Huijun Liu[a, b*]

[a] School of Environmental Science and Engineering, Zhejiang Gongshang University, Hangzhou, Zhejiang 310018, China.

[b]Instrumental analysis center of Zhejiang Gongshang University, Hangzhou 310018, Zhejiang Province, China.

.

*Corresponding author.

Tel.: +34 934024494; fax: +34 934024495.

E-mail address: lhj@zjgsu.edu.cn (H.J. Liu)

Caption

Table. S1 Artificial culture medium.

Table. S2 The value of EC_{50} in *S. obliquus* in different culture times.

Table. S3 The chlorophyll fluorescence parameters of *S. obliquus* treated with enrofloxacin.

Table S1 Artificial culture medium

Compounds	Concentration
$NaHCO_3$	0.10 g/L
$MgSO_4•H_2O$	0.08 g/L
$ZnSO_4•7H_2O$	0.03 g/L
（$NH_4)_2•SO_4$	0.20 g/L
KCl	0.025 g/L
$FeCl_3$, 1%（W/W）	150 μL
Soil extract	500 μL

Table S2 The value of EC_{50} in *S. obliquus* in different culture times

Time（h）	EC_{50}(μg/L)	A_1	A_2	*p*	R^2
24	119.74	-1.8766	111.5462	1.0945	0.99812
48	53.09	6.2716	95.1479	1.6268	0.09975
72	64.37	-2.4995	64.3713	2.2870	0.9959
96	52.64	1.0488	52.6140	1.6675	0.9991

Table S3 The chlorophyll fluorescence parameters of *S. obliquus* treated with enrofloxacin

Time （h）	Dosage （μg/L）	F_0	F_m	Fv/F_m	F_v/F_0
48	0	0.083±0.002	0.198±0.005	0.581±0.004	1.389±0.020
	10	0.118±0.001	0.208±0.002	0.432±0.002	0.760±0.003
	50	0.156±0.003	0.263±0.004	0.480±0.002	0.686±0.006
	80	0.188±0.007	0.304±0.011	0.407±0.002	0.615±0.000
	120	0.267±0.010	0.362±0.013	0.381±0.001	0.355±0.003
	180	0.345±0.003	0.407±0.003	0.261±0.002	0.182±0.003
96	0	0.088±0.001	0.232±0.005	0.613±0.002	1.619±0.069
	10	0.143±0.001	0.305±0.001	0.536±0.001	1.143±0.016
	50	0.208±0.003	0.318±0.008	0.379±0.002	0.595±0.017
	80	0.236±0.001	0.349±0.000	0.365±0.002	0.552±0.023
	120	0.317±0.002	0.375±0.003	0.107±0.002	0.100±0.005
	180	0.371±0.006	0.385±0.004	0.000±0.000	0.033±0.005
